# Association of diet quality and quantity with the risk of sarcopenia based on the Chinese diet balance index 2022

**DOI:** 10.3389/fnut.2025.1562362

**Published:** 2025-05-27

**Authors:** Xiao Yu, Mei Zhang, Yongjun Wang, Lianlong Yu, Changsheng Zhao

**Affiliations:** ^1^Department of Clinical Nutrition, The Second Hospital, Cheeloo College of Medicine, Shandong University, Jinan, China; ^2^Department of Medical Clinical Laboratory, The People’s Hospital of Huaiyin Jinan, Jinan, China; ^3^Department of Clinical Nutrition, The First Affiliated Hospital of Shandong First Medical University, Shandong Provincial Qianfoshan Hospital, Jinan, China; ^4^Health Management Institute, Shandong Center for Disease Control and Prevention, Jinan, China

**Keywords:** older adults, Dietary Balance Index 2022, sarcopenia, red meat and products, poultry and game, cereal

## Abstract

**Objective:**

This study aimed to analyze the dietary structure characteristics and the association between food intake and sarcopenia among older adults in China, based on the DBI-22 scoring criteria. This analysis was intended to provide guidance for improving dietary balances and nutritional management related to sarcopenia.

**Methods:**

A stratified random cluster sampling method was employed to select 1,478 elderly individuals aged 60 and above in Chinese. A questionnaire survey was conducted, which included the Sarcopenia-Five (SARF) scale and Food Frequency Questionnaires (FFQ). The China Dietary Balance Index 2022 (DBI-22) was utilized to assess dietary quality. Generalize Linear Model (GLM) and logistic regression analysis were applied to examine dietary factors influencing sarcopenia.

**Results:**

The issue of dietary imbalance among the elderly was found to be severe, with 52.11% of participants exhibiting deficient intake, 46.54% displaying excessive intake, and 59.78% demonstrating overall dietary imbalance (moderate-to-high levels). Screening conducted using the SARF scale revealed a sarcopenia rate of 24.82%. Excessive intake of cereal was identified as a risk factor for sarcopenia (*OR* = 1.490, 95%CI: 1.149, 1.939; *p* = 0.003), whereas increased consumption of red meat and products, poultry and game was found to reduce this risk (*OR* = 0.560, 95%CI: 0.342, 0.886; *p* = 0.016).

**Conclusion:**

The dietary habits of older adults were problematic, with both insufficient and excessive food intake. Too much cereal posed a risk for sarcopenia, whereas more red meat and products, poultry and game served as a protective factor.

## 1 Introduction

China faces a severe aging population issue, with 18.7% being elderly. Nearly half of them suffer from malnutrition, causing health problems due to dietary imbalances. Improving elderly nutrition is now a healthcare priority (1–4). However, many elderly individuals had poor nutritional knowledge and imbalanced diets, leading to increased chronic diseases. Thus, they are a primary focus for nutritional education. Studying their dietary intake can reveal structural diet issues (5–8). Scientific and reasonable dietary recommendations could be proposed to improve their nutritional status, thereby prevent the occurrence and progression of diseases, and reduce societal burdens (1, 2).

Sarcopenia was defined as the gradual loss of muscle mass and function, including muscle strength and performance, primarily affecting individuals over the age of 50 (3, 4). It was estimated that the prevalence of sarcopenia was reported to be up to 29% in older adults in the community healthcare setting (5). Aging inevitably resulted in declines in muscle function with prevalence increasing with age (6, 7). And muscle mass and muscle strength decreased at a rate of 1–2% and 1.5–5% per year, respectively (8). The functional deterioration linked to sarcopenia may result in adverse outcomes, including falls, fractures, and frailty, thereby substantially affecting the quality of life and overall health of older adults (9). Modifications in dietary and lifestyle factors are thought to have a considerable impact on muscle function in the elderly (10).

The Diet Balance Index (DBI) was developed based on the Chinese Dietary Guidelines and the Balanced Diet Pyramid to assess the dietary quality of Chinese people (11, 12). This scoring method utilized food groups as scoring items, eliminating the need for complex calculations of nutrients, and effectively reflected both deficient and excessive intake levels. In 2023, Yuna He et al. modified the existing DBI-16 and established the Chinese Diet Balance Index-22 (DBI-22), which was more suitable for all general populations aged over two years.

Research on dietary factors linked to sarcopenia in the elderly using the DBI-22 is limited globally (13). Dietary factors may influence muscle health and the progression of sarcopenia. The present study hypothesizes that there is a significant association between dietary quality, as measured by the DBI-22, and the incidence or severity of sarcopenia in elderly individuals. To validate this hypothesis and the underlying theory, a random cluster sampling was used to recruit 1,487 seniors aged 60 + in China, collecting demographic data, SARC-F scale results, and Food Frequency Questionnaires. Participants’ food scores were calculated based on DBI-22 criteria. This evaluation aimed to assess the dietary quality of elderly individuals in China, and to explore the dietary factors influencing sarcopenia. It was intended to provide a basis for nutritional intervention and guidance for the elderly population.

## 2 Method

### 2.1 Study participants

From July to October 2024, a representative sample of the general population in Shandong Province was obtained through a multistage proportional stratified cluster sampling method among the elderly population aged 60 and above in 10 regions (5 urban districts and 5 rural counties) of Shandong Province, China. The exclusion criteria included: Individuals with cognitive impairments, severe psychological disorders, or mental illnesses; Individuals with serious chronic diseases, such as heart failure, respiratory failure, cirrhosis, or renal failure; Individuals with chronic wasting diseases, such as malignant tumors, HIV/AIDS, or chronic gastrointestinal diseases; Individuals with severely restricted physical activity or those unable to perform activities of daily living. A total of 1,600 questionnaires were collected. After excluding low-quality responses and those with abnormal energy intake (defined as exceeding 4,000 kcal or below 500 kcal per day), 1,487 valid questionnaires remained. All participants signed informed consent forms.

### 2.2 Outcome variable

The Sarcopenia-Five (SARC-F) scale comprised several assessment items, including strength, assistance in walking, rising from a chair, climbing stairs, and falls, along with calf circumference (4). Each item was assigned a score ranging from 0 to 2, based on the difficulty of completion. Calf circumference was evaluated as either normal or abnormal, receiving scores of 0 or 10, respectively. The total score thus ranged from 0 to 20, with a score of ≥11 indicating a positive screening result for sarcopenia.

*Measurement of height and weight*: Participants were instructed to remove their coats, shoes, and hats, minimizing clothing as much as possible, before standing on the height-weight measuring device. The measurements were conducted by trained community doctors, with height recorded to the nearest 0.1 cm and weight to the nearest 0.1 kg.

*Grip strength assessment*: Using the grip dynamometer (Cat#: EH101, CAMRY, Guangzhou, China), subjects adopted a standing position with their bodies upright and arms hanging naturally. They were asked to hold the dynamometer with their dominant hand, with the palm facing inward and the dial facing outward. The dynamometer was ensured to have no contact with the body or clothing. Each subject was instructed to exert their maximum force during testing. The dominant hand was tested once, followed by a 30-s rest period before a second measurement was taken. The maximum value of the two measurements was recorded.

*Gait speed measurement*: A 4-meter straight line was marked at the measurement site. A stopwatch was used to measure the time taken by the participants to walk the distance under normal conditions. The test was repeated twice, and the shorter time was included in the analysis. A gait speed of ≥0.8 m/s was considered normal.

DBI-2022 comprises 14 subgroups of 8 components from the Dietary Guidelines for Chinese residents, including: (1) cereal; (2) vegetable; (3) fruit; (4) dairy; (5) soybean; (6) red meats/products/poultry/game; (7) fish/shrimp; (8) egg; (9) cooking oils; (10) alcoholic beverage; (11) added sugar; (12) addible salt; (13) diet variety; and (14) drinking water. A score of 0 for each DBI-22 component means that the individual has reached the recommended intake amounts of the corresponding food group. Positive scores (ranging 1 to 12) indicate the excessive intake level of cereals, red meat/products/poultry/game, eggs, cooking oils, alcoholic beverage, added sugar, salt. While negative scores (ranging −12 to −1) indicate the inadequate intake level of cereals, vegetables, fruits, dairy, soybeans, red meat/products/poultry/game, fish/shrimps, eggs, diet variety, and drinking water. Considering the difference of nutrient requirements in energy consumption, the scoring of these 14 food subgroups was based on the levels of energy intake. The Dietary Balance Index (DBI) evaluation metrics were as follows. Low Bound Score (LBS): This indicated the absolute value of the sum of negative scores from all food categories, reflecting the extent of insufficient intake, with a range of 0 to 72. High Bound Score (HBS): This represented the sum of positive scores from all food categories, reflecting the extent of excessive intake, with a range of 0 to 38. Diet Quality Distance (DQD): This was the sum of the absolute values of scores across all food categories, reflecting the degree of dietary imbalance, with a range of 0 to 90. Total Score (TS): This represented the sum of scores across all food categories, reflecting the average level of dietary quality, with a range of −72 to 38 (11).

Evaluation of food intake quality were categorized as follows: scores between −2 and 2 (with cereal score ranging from −4 to 4 and drinking water and diet variety ranging from −4 to 0) were considered relatively appropriate; scores below −2 indicated insufficient intake, while scores above 2 indicated excessive intake. Evaluation of diet quality were categorized as follows: The scores of LBS, HBS, and DQD were evaluated such that a score of 0 indicated no issues, scores ≤20% indicated appropriate intake, scores between 20 and 40% indicated low levels of intake imbalance, scores between 40 and 60% indicated moderate intake imbalance, and scores exceeding 60% indicated high levels of intake imbalance.

### 2.3 Exposure

The food frequency questionnaire was administered by rigorously trained interviewers to assess the food intake of elderly individuals over the past year through face-to-face evaluations. The design of this questionnaire was primarily based on previous dietary pattern studies conducted abroad and semi-quantitative food frequency questionnaires in China (12, 14), while also considering foods and food categories identified in previous research to be associated with chronic diseases in the elderly population. The questionnaire underwent reliability and validity testing, with correlation coefficients for repeated measurements spaced two weeks apart ranging from 0.56 to 0.87. Correlation coefficients with food intake from the food-frequency questionnaire (FFQ146) ranged from 0.31 to 0.72, and those with dietary records (DR) intake were all between 0.37 and 0.65. All results were statistically significant (*p* < 0.01) (15). The survey included inquiries regarding the consumption of cereals, vegetables, fruits, dairy, soybeans, red meat and products, poultry and game, fish and shrimp, eggs, alcoholic beverages, cooking oil, addible salt, diet variety, and drinking water.

### 2.4 Covariates

Various potential confounders were considered in this analysis. Socio-demographic factors included age, gender (‘Male’ and ‘Female’), residence (‘Rural’, and ‘City’), education level (‘Illiterate’, ‘Primary school’, ‘Junior High School’ and ‘High School and Above’), income, and marriage (‘Married’ and ‘Other’). Health variable included Body Mass Index (BMI).

### 2.5 Statistical analysis

The database was constructed utilizing Epidata 3.1 software, incorporating double data entry by two independent individuals, followed by consistency verification. Food scores were computed based on the criteria outlined in the DBI-22. Variations in variables were evaluated using Student’s t-test or One-Way ANOVA. To examine the association between various food items and the SARF score, as well as sarcopenia, Generalized Linear Models (GLM) and logistic regression analyses were employed. All effected were presented as *β* or OR, with corresponding 95% confidence intervals (CIs). To verify the robustness of the results, sensitivity analysis was performed, and stratified analyses were applied. Restricted Cubic Spline (RCS) were employed to fit the relationship curve between different food score and SARF score as well as sarcopenia. The above analysis was performed using the R (version 4.4.0). *p*-value <0.05 was considered statistically significant.

## 3 Results

### 3.1 Descriptive statistics of diet quality

A total of 1,487 elderly individuals were included in the study after the exclusion of samples with inadequate data quality. The mean age of the participants was 70.21, and 53.19% were female. Overall, the dietary quality of elderly individuals in the Shandong region of China was found to be concerning. For foods with deficient intake, the mean LBS was 29.28 (SD = 6.74), with 52.11% of elderly participants exhibiting moderate to high intake deficiencies. In contrast, only 15% of participants had HBS values within the acceptable range for foods with excessive intake, with a total moderate-to-high excess prevalence of 46.54%. Almost all elderly respondents displayed imbalanced dietary intake, with moderate (39.07%) and high (20.71%) imbalances being prevalent, totaling 59.78% for moderate-to-high imbalance (Table 1). Elderly individuals aged less than 70 years, classified as overweight (24.0 ≤ BMI < 28.0 kg/m^2^), married, residing in urban areas, with an education level of high school or above, employed in stable professions such as civil servants, healthcare workers, and teachers, cohabitating with partners, and earning a monthly income exceeding 1,500 yuan exhibited lower levels of LBS, HBS, and DQD. This group demonstrated relatively lower degrees of dietary imbalance. Additionally, female participants showed lower levels of LBS, while male participants had lower HBS levels (*p* < 0.05), which indicated a greater prevalence of deficient intake among women and excessive intake among men. No significant differences in DQD levels were observed between genders (Table 2).

**Table 1:** The distribution of diet quality for the elderly.

Type	Index	Score	No issue (%)	Suitable (%)	Low (%)	Moderate (%)	High (%)
Deficiency	LBS	29.28 ± 6.74	0 (0.00)	89 (5.99)	623 (41.90)	606 (40.48)	173 (11.63)
Excess	HBS	10.29 ± 4.67	16 (1.08)	220 (14.79)	559 (37.59)	489 (32.89)	203 (13.65)
Imbalance	DQD	39.56 ± 9.93	0 (0.00)	109 (7.33)	489 (32.89)	581 (39.07)	308 (20.71)

LBS, Low Bound Score; HBS, High Bound Score.

**Table 2:** The basic characteristics of participants.

Characteristics	*N* (%)	LBS	*p*	HBS	*p*	DQD	*p*	TS	*p*
Age									
<70	770 (51.78)	28.65 ± 6.69	<0.001^***^	10.11 ± 4.45	0.136	38.77 ± 9.72	0.001^**^	−18.52 ± 5.87	0.003^**^
≥70	717 (48.22)	29.95 ± 6.74		10.47 ± 4.89		40.42 ± 10.09		−19.48 ± 6.08	
Gender									
Male	696 (46.81)	28.91 ± 6.60	0.047^*^	10.61 ± 4.59	0.012^*^	39.52 ± 9.60	0.864	−18.30 ± 6.09	<0.001^***^
Female	791 (53.19)	29.60 ± 6.85		10.00 ± 4.72		39.61 ± 10.22		−19.60 ± 5.83	
BMI (kg/m^2^)									
<18.5	46 (3.09)	32.70 ± 7.72	<0.001^***^	11.74 ± 4.61	<0.001^***^	44.43 ± 10.81	<0.001^***^	−20.96 ± 6.71	<0.001^***^
18.5 ~ 24.0	744 (50.03)	29.64 ± 6.51		10.53 ± 4.78		40.17 ± 9.88		−19.11 ± 5.73	
24.0 ~ 28	544 (36.59)	28.31 ± 6.64		9.60 ± 4.42		37.91 ± 9.57		−18.72 ± 5.98	
≥28.0	153 (10.29)	29.92 ± 7.35		11.10 ± 4.69		41.03 ± 10.23		−18.82 ± 6.91	
Marriage									
Married	1,193 (80.23)	28.86 ± 6.56	<0.001^***^	10.00 ± 4.53	<0.001^***^	38.86 ± 9.59	<0.001^***^	−18.85 ± 5.93	0.079
Other	294 (19.77)	30.99 ± 7.19		11.43 ± 5.03		43.42 ± 10.76		−19.56 ± 6.19	
Residence									
City	884 (59.45)	26.93 ± 5.62	<0.001^***^	9.01 ± 4.22	<0.001^***^	35.94 ± 8.37	<0.001^***^	−17.92 ± 5.36	<0.001^***^
Rural	603 (40.55)	32.73 ± 6.77		12.16 ± 4.67		44.88 ± 9.65		−20.57 ± 6.50	
Education									
Illiterate	427 (28.72)	32.54 ± 6.70	<0.001^***^	12.26 ± 4.53	<0.001^***^	44.80 ± 9.64	<0.001^***^	−20.28 ± 6.15	<0.001^***^
Primary School	472 (31.74)	29.75 ± 6.08		10.54 ± 4.45		40.29 ± 9.02		−19.21 ± 5.67	
Junior High School	309 (20.78)	28.03 ± 6.15		9.64 ± 4.14		37.67 ± 8.53		−18.39 ± 6.08	
High School and above	279 (18.76)	24.87 ± 5.64		7.56 ± 4.43		32.43 ± 8.25		−17.32 ± 5.69	
Occupation									
Farmer	995 (66.91)	29.30 ± 6.79	0.846	10.46 ± 6.79	0.024^*^	39.76 ± 6.79	0.353	−18.85 ± 6.79	0.048^*^
Mobile service industry	211 (14.19)	29.31 ± 6.53		10.29 ± 6.53		39.60 ± 6.53		−19.02 ± 6.53	
Fixed industry	243 (16.34)	28.74 ± 6.72		9.61 ± 6.72		38.36 ± 6.72		−19.13 ± 6.71	
Other	38 (2.56)	31.87 ± 6.53		10.11 ± 6.53		41.97 ± 6.53		−21.76 ± 6.53	
Live with partner									
Yes	1,096 (73.71)	28.50 ± 6.48	<0.001^***^	9.81 ± 4.48	<0.001^***^	38.31 ± 9.50	<0.001^***^	−18.69 ± 5.83	0.002^**^
No	391 (26.29)	31.45 ± 6.97		11.61 ± 4.93		43.07 ± 10.29		−19.84 ± 6.33	
Income									
<500 yuan	424 (28.51)	33.32 ± 6.22	<0.001^***^	12.72 ± 4.33	<0.001^***^	46.04 ± 8.94	<0.001^***^	−20.60 ± 5.91	<0.001^***^
500–1,000 yuan	397 (26.70)	29.76 ± 6.24		10.79 ± 4.27		40.54 ± 8.76		−18.97 ± 6.13	
1,000–1,500 yuan	263 (17.69)	28.11 ± 5.80		9.75 ± 4.09		37.86 ± 8.30		−18.37 ± 5.64	
>1,500 yuan	403 (27.10)	25.32 ± 5.74		7.59 ± 4.24		32.91 ± 8.28		−17.73 ± 5.79	
The score of SARC-F									
0 ~ 10	1,118 (75.18)	29.03 ± 6.66	0.013^*^	10.53 ± 4.56	<0.001^***^	39.56 ± 9.82	0.959	−18.49 ± 5.82	<0.001^***^
11 ~ 20	369 (24.82)	30.05 ± 6.92		9.54 ± 4.92		39.59 ± 10.27		−20.50 ± 6.24	

^*^p < 0.05, ^**^p < 0.01, ^**^p < 0.001.

### 3.2 The distribution of DBI-22 composition index score

**Figure 1:**
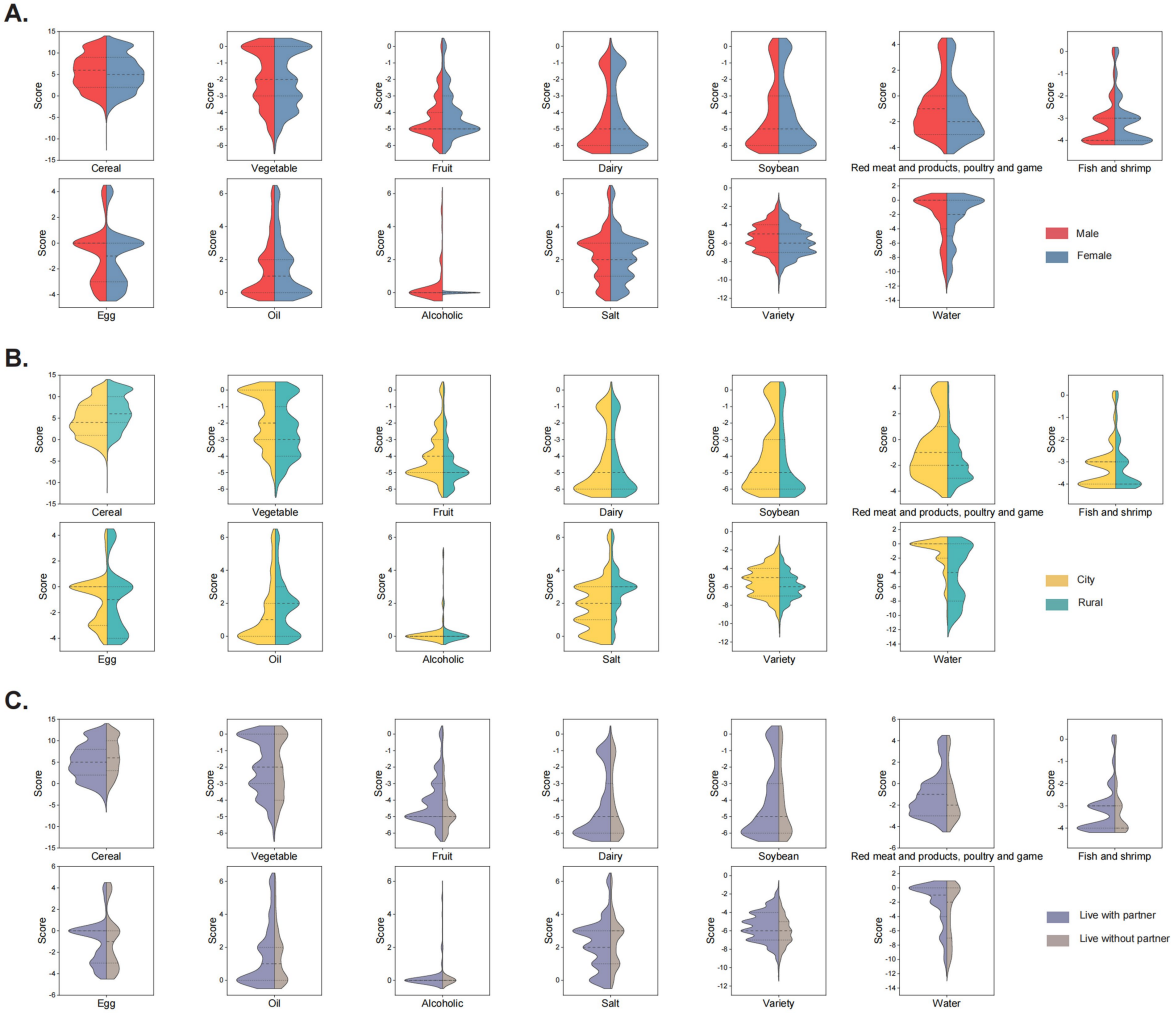
Split violin plot of scores for different food intake. **(A)** Grouped by gender. **(B)** Grouped by residence. **(C)** Grouped by living with a partner or not.

The distribution of DBI-22 scores for different food categories is presented in Table 3. Over 50% of elderly participants in Shandong region of China were found to have excessive intake of cereal-based foods. More than 70% of elderly individuals experienced severe deficiencies in the average daily intake of fruits, dairy products, fish and shrimp and soybeans, with a low diversity of diet variety, all failing to meet the recommended intake levels by the Chinese Dietary Guidelines. In contrast, the intake of vegetables, red meat and products, poultry and game, eggs, cooking oil, and drinking water was found to be relatively satisfactory. Most elderly individuals maintained an appropriate salt intake, although a considerable proportion, amounting to 46.27%, also exhibited excessive intake (Tables 3, 4). The comparative chart of food intake scores reflected the food selection preferences of different populations. Overall, the distribution of food intake scores among males and females was found to be similar. Both groups demonstrated excessive intake of cereal, while deficiencies in the intake of fruits, dairy products and fish and shrimp were prominent. Female participants had lower median scores for red meat and products, poultry and game intake and dietary diversity, with significantly less alcohol consumption observed. Compared to elderly individuals residing in urban areas, participants from rural areas had higher median scores for cereal, cooking oil, and salt intake, alongside lower consumption of vegetables, fruits, red meat and products, poultry and game, fish and shrimp, eggs, drinking water, and dietary diversity. Furthermore, elderly individuals living without partner exhibited higher median scores for cereal intake compared to those cohabiting, while lower scores were noted for red meat and products, poultry and game and egg intake, with minor differences observed for other food categories (Figure 1).

**Table 3:** Distributions of scores for the DBI-22 components and the percentages of participants with each score.

Scores	Cereal	Dairy	Egg	Red meat and products, poultry and game	Fish and shrimp	Soybean	Vegetable	Fruit	Alcoholic	Water	Oil	Salt	Variety
−12	0.07									0.27			
−11										1.08			0.20
−10										4.24			0.47
−9										3.30			2.29
−8										2.82			8.07
−7										8.41			20.85
−6	0.07	44.18				37.86	0.81	11.03		2.62			22.66
−5	0.07	17.96				18.56	5.72	43.31		2.62			20.91
−4	0.40	9.62	15.40	5.99	49.70	11.43	16.01	20.98		7.26			15.80
−3	0.87	4.91	18.49	24.28	33.36	10.69	20.17	11.50		0.74			6.86
−2	1.61	5.18	10.56	21.12	9.41	3.30	17.15	8.47		18.29			1.68
−1	3.16	17.08	3.50	16.21	2.82	5.51	9.48	1.28		0.61			0.20
0	8.68	1.08	42.50	14.19	4.71	12.64	30.67	3.43	91.46	47.75	42.97	15.33	
1	4.98		0.27	4.44					2.02		13.18	18.16	
2	7.40		0.54	2.82					3.36		20.58	20.24	
3	8.47		2.02	4.30					0.40		8.54	32.89	
4	8.34		6.72	6.66					0.81		5.85	8.20	
5	6.99								1.55		3.63	1.55	
6	9.35								0.40		5.25	3.63	
7	6.79												
8	6.99												
9	8.00												
10	3.09												
11	3.30												
12	11.37												

**Table 4:** The distribution of DBI-22 composition index score.

Type	Cereal (%)	Vegetable (%)	Fruit (%)	Dairy (%)	Soybean (%)	Red meat and products, poultry and game (%)	Fish and shrimp (%)	Egg (%)	Oil (%)	Alcoholic (%)	Salt (%)	Variety (%)	Water (%)
Adequacy	653 (43.91)	852 (57.30)	197 (13.25)	347 (23.34)	319 (21.45)	874 (58.78)	252 (16.95)	853 (57.36)	1,141 (76.73)	1,440 (96.84%)	799 (53.73)	365 (24.55)	1,110 (74.65)
Deficiency	3 (0.20)	635 (42.70)	1,290 (86.75)	1,140 (76.66)	1,168 (78.55)	450 (30.26)	1,235 (83.05)	504 (33.89)	-	-	-	1,122 (75.45)	377 (25.35)
Excess	831 (55.88)	-	-	-	-	163 (10.96)	-	130 (8.74)	346 (23.27)	47 (3.16)	688 (46.27)	-	-

### 3.3 Dietary influencing factors of sarcopenia

**Figure 2:**
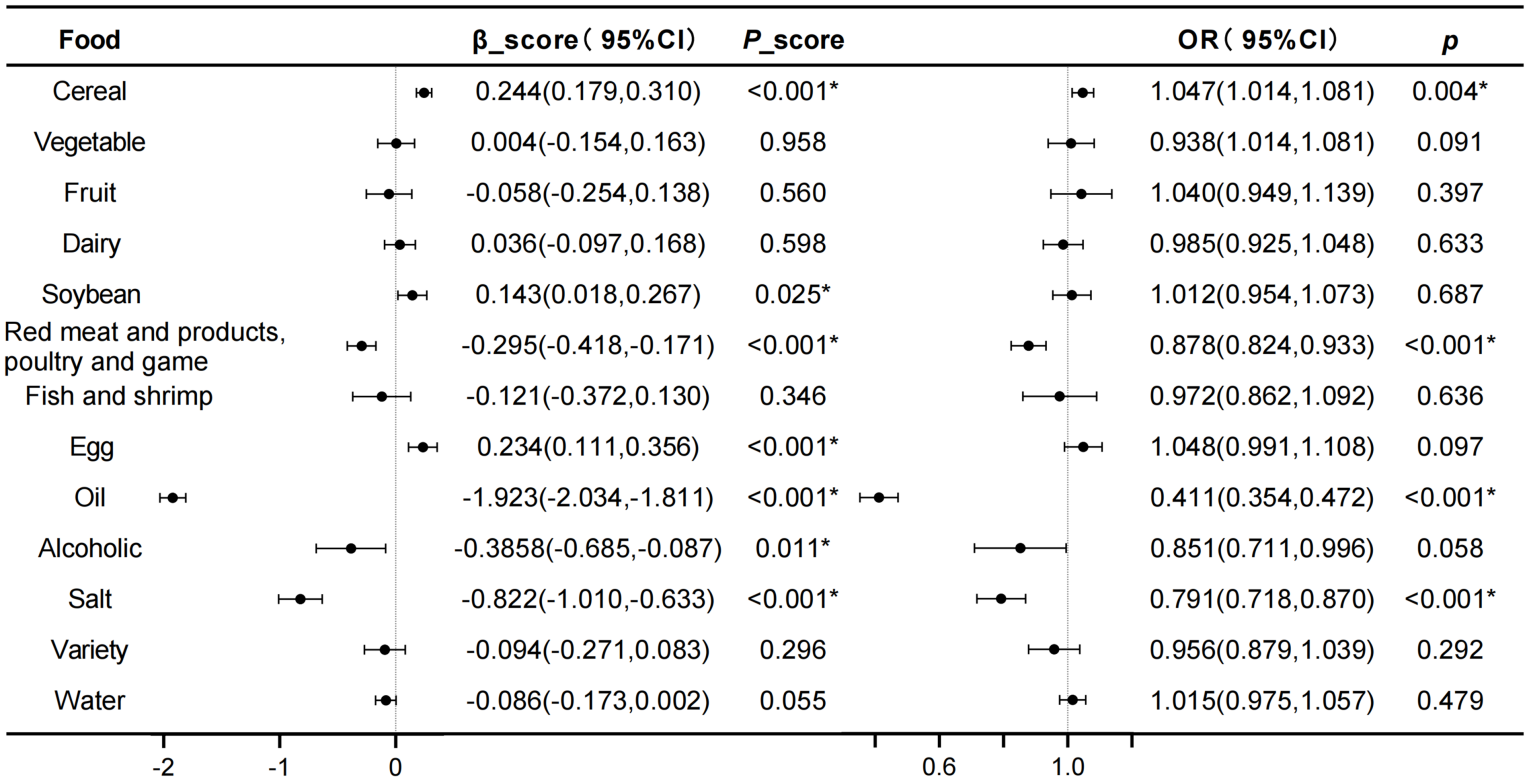
Analysis of the correlation between scores of different food intake calculated based on DBI-22 and SARF score, as well as sarcopenia, using GLM and logistic regression methods. The model was fully adjusted for age, gender, BMI, income, residence, marriage, education level. ^*^p < 0.05.

A screening conducted through the SARF scale revealed an illness rate of sarcopenia at 24.82% (369/1487 participants) (Table 2). According to the results of GLM analysis, the intake scores of cereal and eggs were found to be positively correlated with the SARF scores (*β* > 0). Conversely, the intake of red meat and products, poultry and game, cooking oil, alcohol, and salt exhibited negative correlations with the SARF scores (*β* < 0). Logistic regression analysis showed that cereal intake was associated with an increased risk of developing sarcopenia (*OR* = 1.047, 95%*CI*: 1.014, 1.081. *p* = 0.004), while Red meat and products, Poultry and game (*OR* = 0.878, 95%*CI*: 0.824, 0.933. *p* < 0.001), cooking oil (*OR* = 0.411, 95%*CI*: 0.354, 0.472. *p* < 0.001), and salt (*OR* = 0.791, 95%*CI*: 0.718, 0.870. *p* < 0.001) appeared to lower the risk of sarcopenia (Figure 2).

**Figure 3:**
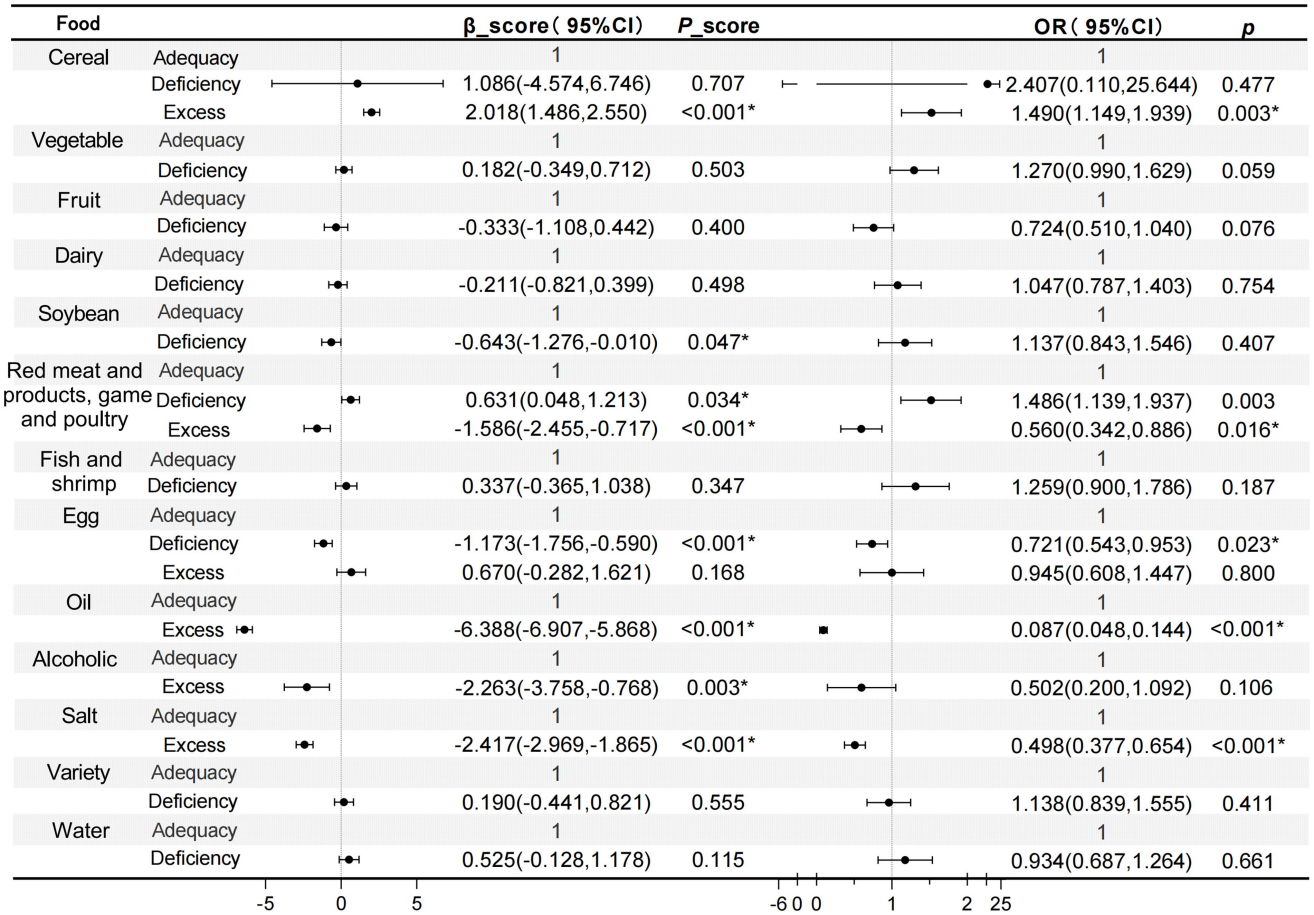
Analysis of the correlation between diet quality of different food intake calculated based on DBI-22 and SARF score, as well as sarcopenia, using GLM and logistic regression methods. The model was fully adjusted for age, gender, BMI, income, residence, marriage, education level. ^*^p < 0.05.

Further analysis of dietary food intake quality in relation to SARF score and sarcopenia revealed that excessive consumption of cereal foods constituted a risk factor for sarcopenia, whereas excessive intake of red meat and products, poultry and game served as a protective factor. Additionally, it was observed that excessive consumption of cooking oil and salt could also reduce the risk of sarcopenia (Figure 3). Stratified analyses based on characteristics were performed to investigate whether food intake was related to the risk of sarcopenia in different populations. It was found that in individuals who aged 70 years and above, males, urban residents, and cohabiting without partner, the cereal intake scores were associated with an increased risk of sarcopenia. In contrast, for individuals under 70 years of age, females, rural residents, or elderly individuals cohabiting with a partner, the influence of cereal intake on sarcopenia was deemed insignificant (*p* < 0.05). In individuals under 70 years of age residing in urban areas and cohabiting with a partner, vegetable intake was observed to lower the risk of sarcopenia. Across all population groups, red meat and products, poultry and game consumption consistently demonstrated a protective effect against sarcopenia, with stable results observed. Furthermore, cooking oil and salt intake were also associated with low *OR* value regarding sarcopenia (Table 5).

**Table 5:** Stratified analysis of the association between DBI-22 composition and sarcopenia.

Characteristics	Cereal (*p*)	Vegetable (*p*)	Fruit (*p*)	Dairy (*p*)	Soybean (*p*)	Red meat and products, poultry and game (*p*)	Fish and shrimp (*p*)	Egg (*p*)	Oil (*p*)	Alcoholic (*p*)	Salt (*p*)	Variety (*p*)	Water (*p*)
Age													
<70	1.024 (0.343)	0.885 (0.042^*^)	0.945 (0.479)	0.933 (0.191)	1.086 (0.074)	0.798 (<0.001^***^)	0.835 (0.101)	1.079 (0.120)	0.360 (<0.001^***^)	0.656 (0.030^*^)	0.865 (0.054)	1.011 (0.861)	0.999 (0.975)
≥70	1.066 (0.003^**^)	0.983 (0.741)	1.085 (0.170)	1.010 (0.800)	0.961 (0.310)	0.921 (0.033^*^)	1.057 (0.465)	1.028 (0.426)	0.439 (<0.001^***^)	0.939 (0.533)	0.747 (<0.001^*^)	0.902 (0.069)	1.019 (0.477)
Gender													
Male	1.043 (0.077)	0.923 (0.152)	0.920 (0.283)	0.964 (0.448)	1.002 (0.973)	0.855 (0.001^**^)	1.005 (0.955)	1.056 (0.198)	0.538 (<0.001^***^)	0.858 (0.079)	0.841 (0.016^*^)	0.951 (0.424)	0.945 (0.063)
Female	1.053 (0.021^*^)	0.950 (0.334)	1.128 (0.042^*^)	0.996 (0.922)	1.017 (0.679)	0.893 (0.008^**^)	0.930 (0.393)	1.039 (0.330)	0.270 (<0.001^***^)	<0.001 (0.983)	0.753 (<0.001)	0.951 (0.398)	1.087 (0.007)
Residence													
City	1.065 (0.003^**^)	0.850 (0.002^**^)	1.011 (0.859)	0.944 (0.186)	1.063 (0.127)	0.873 (<0.001^***^)	0.927 (0.398)	1.134 (0.008^**^)	0.287 (<0.001^***^)	0.868 (0.212)	0.7800 (<0.001^***^)	1.057 (0.317)	1.018 (0.609)
Rural	1.033 (0.203)	1.056 (0.334)	1.090 (0.238)	1.051 (0.304)	0.941 (0.196)	0.875 (0.030^*^)	1.007 (0.932)	0.998 (0.950)	0.479 (<0.001^***^)	0.817 (0.128)	0.786 (0.002^**^)	0.823 (0.004^**^)	1.008 (0.760)
Live with partner													
Yes	1.034 (0.089)	0.906 (0.036^*^)	1.068 (0.228)	0.961 (0.309)	1.008 (0.827)	0.863 (<0.001^***^)	0.955 (0.550)	1.056 (0.154)	0.384 (<0.001)	0.875 (0.163)	0.822 (<0.001^***^)	0.994 (0.914)	1.029 (0.294)
No	1.084 (0.006^**^)	0.998 (0.973)	0.950 (0.589)	1.030 (0.607)	1.013 (0.820)	0.898 (0.039^*^)	1.051 (0.626)	1.044 (0.317)	0.444 (<0.001^***^)	0.759 (0.162)	0.704 (<0.001^***^)	0.880 (0.106)	0.983 (0.639)

^*^p < 0.0, ^**^p < 0.01, ^***^p < 0.001.

**Figure 4:**
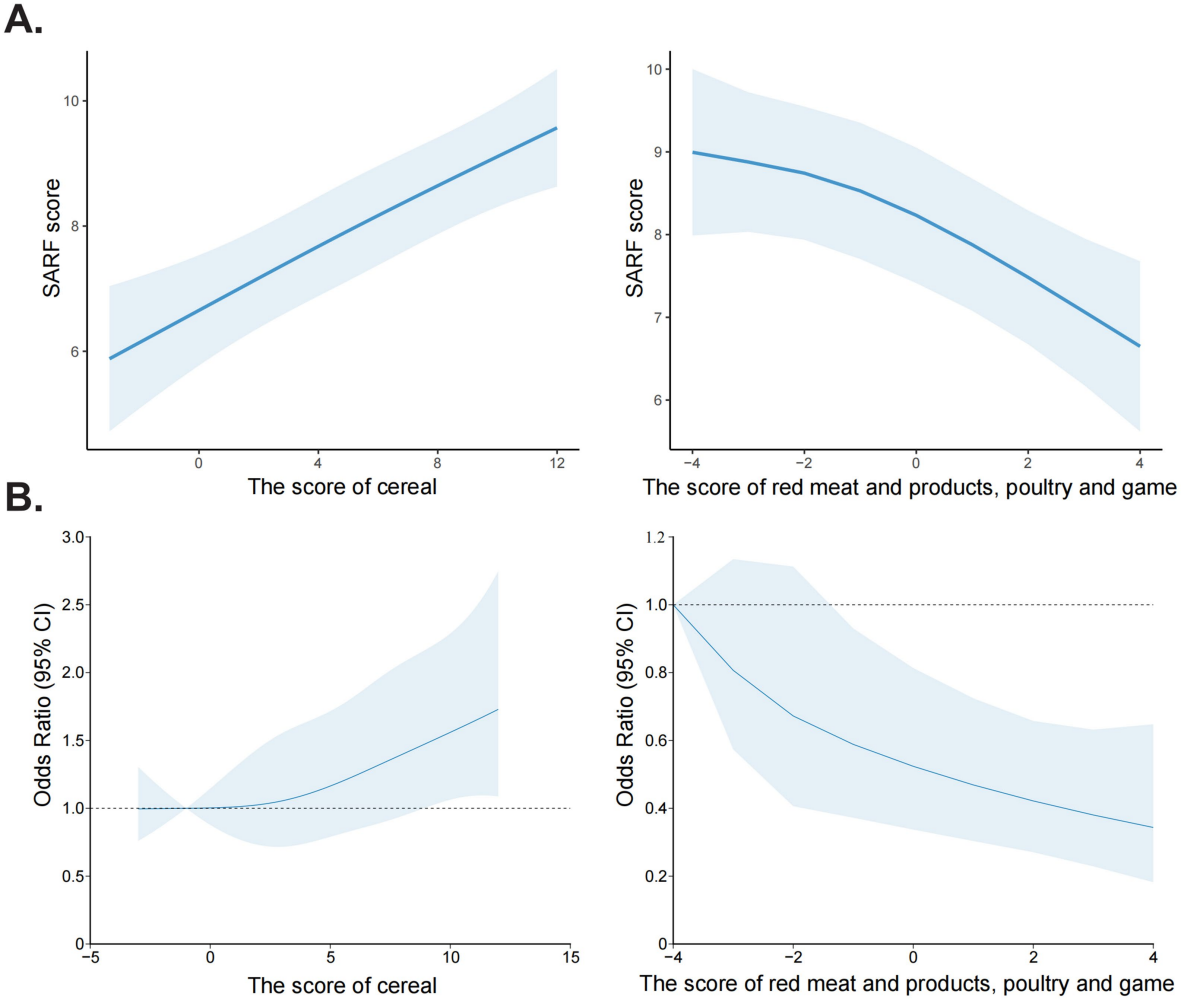
Restricted Cubic Spline (RCS) analysis of the association between food group scores and sarcopenia risk. **(A)** The association between cereal score, red meat and products, poultry and game score with SARF score. **(B)** The association between cereal score, red meat and products, poultry and game score with sarcopenia risk.

The significant portion of dietary food intake attributed to cereal and red meat and products, poultry and game led to a focused influence of these two food groups on sarcopenia. The relationship between these two foods and SARF score, as well as sarcopenia was analyzed using RCS analysis. With the increase in cereal score, both the SARF score and the risk of sarcopenia were found to increase. Conversely, as the red meat and products, poultry and game score increased, a decrease in the SARF score and the risk of sarcopenia was observed (Figure 4).

## 4 Discussion

This study used the DBI-22 to assess the diets of elderly individuals in Shandong, China, and applied the SARF scale to identify sarcopenia and its dietary influences. Many elderly participants had an imbalanced diet, with too much cereal and too little fruit, dairy, soybeans, and fish and shrimp. Adjusted GLM and logistic regression analyses revealed that cereal and red meat and products, poultry and game consumption were linked to sarcopenia. Specifically, excessive cereal intake was found to increase the risk of developing sarcopenia, while high red meat and products, poultry and game consumption was observed to decrease this risk.

The elderly in Shandong Province, China, mainly rely on cereal, with over 70% consuming insufficient fruits, dairy, soybeans, and fish and shrimp, leading to low diet diversity, which contrasts with the Chinese Dietary Guidelines. According to the 2022 Chinese Dietary Guidelines and relevant scientific studies, adults were advised to consume between 200 to 350 grams of fruit daily (16). However, investigations revealed that many elderly individuals reported fruit intake significantly below this standard. Additionally, a lack of dietary habits involving dairy products was noted, where the recommended consumption was 300 to 400 mL of milk or equivalent protein-rich dairy products each day, essential for supporting bone health and overall nutritional status. The survey indicated that the dairy intake among the elderly population was far below the recommended levels. Soybean consumption fell short of the 15 g daily recommendation. Likewise, many elderly individuals did not meet the advised 300 to 500 g weekly intake of fish and shrimp, despite its health benefits (17).

The imbalanced dietary structure observed among the elderly population could result in significant health issues and burdens (18, 19). Internationally recognized dietary structures, which are deemed suitable for the physiological health of older adults, typically emphasize the need for diversity and balanced intake of various nutrients (20, 21). For instance, Mediterranean diet patterns recommended by the European Society of Cardiology highlighted the importance of increasing the consumption of fruits, vegetables, whole cereal, low-fat dairy products, lean meats, and fish and shrimp, while concurrently limiting the intake of processed foods, red meat and products, poultry and game, and sugar-sweetened beverages (13, 22–24). Deficient fruit intake was associated with potential deficiencies in nutrients such as vitamin C and dietary fiber, which could increase the risks of constipation and cardiovascular diseases (25, 26). The low consumption of dairy and soybean products was likely to lead to deficiencies in calcium and protein, adversely affecting bone health and immune function (27–29). Furthermore, inadequate fish and shrimp consumption may have reduced the intake of unsaturated fatty acids, thereby heightening the risk of cardiovascular and cerebrovascular diseases (30). The low dietary diversity could contribute to an overall imbalance in nutrition, exacerbating health problems among the elderly (31).

Proper nutrition is key to delaying muscle aging, with a strong link between diet and sarcopenia (32). Muscle loss is also an independent risk factor for osteoporosis, leading to higher mortality rates in the elderly (33). The prevalence of sarcopenia among older adults has demonstrated a concerning upward trend (34). In our survey, 24.82% of the elderly population screened were diagnosed with sarcopenia. The pathogenesis of sarcopenia was noted to be highly complex, involving a confluence of factors such as age, nutrition, immune function, hormones, metabolism, oxidative activity, neurodegenerative diseases, and lifestyle choices (5, 35). Among the nutritional factors, the intake of cereal and red meat and products, poultry and game exhibited a notable impact on the developing of sarcopenia (36–38). It was found that excessive cereal consumption increased the risk of developing sarcopenia (OR = 1.49), while higher red meat and products, poultry and game intake was identified as a protective factor against sarcopenia (OR = 0.56). Since cereal was a staple food, their consumption directly influenced energy supply and the balance of nutrients. Overconsumption of cereal was associated with inadequate intake of other nutrients, particularly high-quality proteins, thereby accelerating muscle degeneration and atrophy. While red meat and products, poultry and game was characterized as a rich source of high-quality protein and iron, providing essential nutrients for older adults, which contributed to maintaining muscle quality and function, supporting muscle synthesis and repair, and promoting overall muscle health (39, 40). On the other hand, the consumption of red meat and products, poultry, and game has been associated with adverse health effects. High intake of red meat, particularly processed varieties, has been linked to increased risks of cardiovascular disease, certain cancers, and obesity (41, 42). High intake of red meat, particularly processed varieties, has been associated with increased risks of cardiovascular disease, certain cancers, and obesity. A systematic review and meta-analysis of prospective studies demonstrated that high red meat consumption was positively correlated with the risk of breast, endometrial, colorectal, colon, rectal, lung, and hepatocellular cancers, while high processed meat intake was linked to elevated risks of breast, colorectal, colon, rectal, and lung cancers. In contrast, our analysis revealed a statistically significant protective association between red meat intake and the outcome of interest (OR = 0.560, 95% CI: 0.342–0.886). Although the observed risk reduction was statistically significant, the relatively wide confidence intervals suggest some uncertainty regarding the precise magnitude of this protective effect. This uncertainty may be partly attributable to the modest sample size (*n* = 1,487), which could reduce the precision of the effect estimate. We suggest that red meat can be part of a healthy dietary pattern if consumed in moderation. Future studies involving larger and more diverse elderly populations are warranted to validate our findings and narrow the confidence intervals, thereby providing a more accurate estimation of the potential protective effect of red meat against sarcopenia.

However, the statistical significance of cooking oil and salt in reducing the risk of sarcopenia was found, which seem to be somewhat perplexing. Scholars in Japan showed that the risk of sarcopenia with decreased muscle mass and strength was positively related to the amount of salt intake, and efforts to reduce salt intake may prevent sarcopenia (43). The specific mechanisms by which these factors contribute to sarcopenia require further investigation. The sources of salt and oil intake in the Chinese population differ markedly from Western dietary patterns, where processed foods serve as the primary contributors. In contrast, added salt and cooking oils constitute the predominant sources in Chinese diets, which may lead to distinct implications for health outcomes. Ultra-processed foods (UPFs), characterized by extensive industrial processing and high content of additives, sugars, oil and salt, demonstrate a significant dose–response relationship with elevated risk of low muscle mass in adults. Limiting UPF consumption could represent a viable intervention strategy for preserving muscle mass in middle-aged and younger populations, with potential benefits for maintaining physical function during aging (44). In this study, salt and oil intake were assessed at the household level using FFQ-based monthly estimates, which may introduce measurement bias. The assessment did not include other sources such as processed/pre-packaged foods, restaurant meals, or workplace canteens. Additionally, the distribution of household consumption may not accurately reflect the intake of individual participants, particularly older adults. The types of salt and oil consumed (e.g., animal vs. plant oil, iodized vs. non-iodized salt) were also not recorded, potentially leading to misclassification. The unexpected inverse association between salt/oil intake and sarcopenia risk may be affected by residual confounding (e.g., physical activity, total energy intake, or dietary patterns involving fermented or preserved foods). The observed inverse association between salt/oil intake and sarcopenia risk may be influenced by residual confounding factors (e.g., physical activity, total energy intake, or dietary patterns involving fermented/preserved foods). Future studies should incorporate more detailed individual-level dietary assessments, including the sources and types of cooking oils and salt, while accounting for culinary practices and cultural contexts, and explore interactions with dietary patterns. These refined measurements of oil/salt consumption would enable more robust investigations of their associations with sarcopenia.

Proper diet is vital for preventing and managing sarcopenia (13). It’s advised to optimize dietary habits with a focus on diverse and balanced foods, including moderate amounts of fish and shrimp, poultry, eggs, lean meats, dairy, and soy for sufficient high-quality protein intake. Furthermore, an increase in the consumption of vegetables and fruits was suggested to provide abundant antioxidant nutrients and vitamins, which play a role in maintaining muscle health. However, we did not recommend excessive cooking oil and salt intake in the elderly diet to avoid sarcopenia since their unfavorable risks for hyperlipidemia and hypertension (45, 46).

There were several strengths in this study. Based on the latest DBI-22 scoring criteria, this study analyzed the issues of dietary imbalance among the elderly population in Shandong Province, China, as well as the association between dietary factors and sarcopenia. However, there were also some limitations. One important limitation of this study is the reliance on the SARC-F questionnaire for sarcopenia screening. While the SARC-F is a simple, low-cost, and validated tool recommended for preliminary assessment in large-scale or community-based studies, it is inherently subjective and may result in misclassification due to its lower sensitivity compared to objective measures such as dual-energy X-ray absorptiometry (DEXA) or handgrip strength testing. This may lead to underestimation or overestimation of true sarcopenia prevalence. Therefore, future studies should consider incorporating objective diagnostic criteria to enhance the accuracy of sarcopenia identification. Dietary data are obtained from the scale, and recall bias is inevitable in the elderly. Reliable information on added sugar intake among elderly Chinese populations was not included. This is due to the complexity of dietary patterns and the lack of standardized methods for assessing added sugar consumption in this specific demographic. As such, we faced significant difficulties in accurately quantifying added sugar intake for our study participants. However, the specific mechanisms by which these factors contribute to sarcopenia require further investigation. By excluding individuals with major chronic diseases or cognitive impairments, the study population represents a relatively healthy subset of older adults, which may limit generalizability to medically complex populations. However, this approach was necessary to reduce confounding and enhance the interpretability of diet-sarcopenia relationships in community-dwelling older adults with preserved functional status. Future longitudinal studies incorporating disease-specific dietary assessments and stratified analyses by comorbidity burden are warranted.

Moreover, this study did not incorporate repeated measurements across different seasons or account for seasonal variation in the analytical models. Dietary patterns exhibit distinct seasonal fluctuations, particularly in the consumption of fresh produce (e.g., fruits, vegetables, and aquatic products), which may substantially influence food choices. This effect is especially pronounced among older adults, who typically prioritize seasonal and locally sourced ingredients. Significant seasonal variations in nutrient intake and dietary pattern adherence were observed, with statistically significant differences (*p* < 0.001) in the mean values of HBS, LBS, and DQD across the four seasons (47). A notable limitation of the present study is its reliance on a single administration of the food frequency questionnaire between July and October to examine seasonal effects. While this design ensured standardized timing of data collection across participants, it precluded longitudinal comparisons of dietary pattern stability across seasons. Future nutritional epidemiology research should incorporate seasonal assessments to adequately capture and report seasonal influences on dietary behaviors and health outcomes, such as sarcopenia.

In our study, we primarily focused on the association between dietary factors and sarcopenia, recognizing that sarcopenia represents only one component of the multifactorial process that influences health in aging. Aging is accompanied by a complex array of factors including changes in body composition and deteriorations in cardiometabolic health, which are intricately linked to functional outcomes in older populations. Moreover, we acknowledge that the observed associations may also have implications for related conditions. For instance, changes in body composition such as loss of lean mass and increased adiposity, as well as abnormalities in cardiometabolic parameters, have been consistently linked to adverse health outcomes. In this light, our study’s insights into dietary influences provide a foundation for future research aimed at testing specific hypotheses regarding the roles of various food components in mitigating risks associated with aging. We recommend that future research should expand the scope of investigation to include detailed evaluations of body composition and cardiometabolic health parameters. Additionally, translational studies are warranted to explore how these nutritional insights can be integrated into clinical practices for improved management and prevention of sarcopenia and other age-related conditions. Such efforts could ultimately contribute to tailored dietary recommendations that support healthy aging and longevity.

## 5 Conclusion

The dietary habits of elderly individuals in China, have raised concerns due to issues of both insufficient and excessive food consumption. An overconsumption of cereals has been identified as a potential risk factor for sarcopenia, while a higher intake of red meat and products, poultry and game appears to serve as a protective factor.

## Data Availability

The original contributions presented in the study are included in the article/supplementary material, further inquiries can be directed to the corresponding author.
